# Sustainable Synthesis of Fe^3+^-Responsive Fluorescent Probes from Crab Shell Waste-Derived Chitosan

**DOI:** 10.3390/nano16150933

**Published:** 2026-07-29

**Authors:** Yifan Ren, Jingnan Hu, Huan Chen, Yutong Ye, Ruiqi Zhang, Ruiyu Mi

**Affiliations:** Engineering Research Center of Ministry of Education for Geological Carbon Storage and Low Carbon Utilization of Resources, Beijing Key Laboratory of Materials Utilization of Nonmetallic Minerals and Solid Wastes, National Laboratory of Mineral Materials, Hebei Key Laboratory of Resource Low-Carbon Utilization and New Materials, School of Materials Science and Technology, China University of Geosciences (Beijing), Beijing 100083, China; yifan_ren@email.cugb.edu.cn (Y.R.); 2103230048@email.cugb.edu.cn (J.H.); 3103250008@email.cugb.edu.cn (H.C.); 1003210206@email.cugb.edu.cn (Y.Y.); 2103250054@email.cugb.edu.cn (R.Z.)

**Keywords:** clusterization-triggered emission, Fe^3+^ detection, fluorescent probe, chitosan

## Abstract

It is crucial to develop advanced fluorescence sensing platforms for precise detection of metal pollutants in environmental monitoring. However, traditional fluorescent probes are often limited by aggregation-caused quenching (ACQ). In contrast, clusterization-triggered emission (CTE) probes, based on non-conjugated systems, exhibit excellent photostability by relying on spatial clusterization to restrict molecular motion. Crab shells, an abundant aquatic waste, are rich in chitin (20–30%). Following deacetylation, chitin is converted into chitosan, a biocompatible and biodegradable biopolymer with excellent potential for chemical modification. In this study, a novel chitosan-based CTE fluorescent probe (CS-FA) was synthesized via a facile cross-linking condensation reaction between chitosan and formaldehyde. This process successfully restricts intramolecular motion and promotes the tight clustering of electron-rich heteroatoms, thereby activating the CTE mechanism. The resultant CS-FA probe exhibits strong and stable blue fluorescence and demonstrates high selectivity and sensitivity toward Fe^3+^ in aqueous media. In the concentration range of 10–100 µM, the fluorescence intensity decreases linearly with the Fe^3+^ concentration, yielding a competitive limit of detection (LOD) of 0.52 µM. Mechanistically, the specific coordination between Fe^3+^ and the cross-linked polymer provides a dominant non-radiative decay pathway, leading to significant fluorescence quenching. Ultimately, this work not only proposes an innovative strategy for constructing sensitive and biomass-derived probes for Fe^3+^ monitoring but also broadens the high-value utilization pathways of marine waste, thereby providing a sustainable waste-to-resource strategy.

## 1. Introduction

Iron is an essential trace element involved in hemoglobin synthesis, energy metabolism, and enzymatic reactions, but excess iron can cause oxidative damage and organ disease [1,2]. In the environment, the concentration of iron ions has a critical impact on water quality and soil health; exceeding the standard concentration may cause water discoloration, affect aquatic life, and influence nutrient availability and microbial activity in soils [3,4]. Therefore, it is essential to develop a sensitive, specific, and simple method for detecting Fe^3+^ in environmental and biological systems [5,6].

There are many ways to detect iron ions, including electrochemical methods, atomic absorption spectrometry, colorimetry, and fluorescent probe methods [7,8,9,10]. Among them, the fluorescence method offers high sensitivity, simple operation, and strong specificity, and can be effectively applied to the detection of various ions [11,12,13]. Carbon quantum dots, small-molecule fluorescent probes, and CTE fluorescent materials can all be used to detect iron ions. Kang et al. [14] prepared a new type of green carbon quantum dots (ILB-CQDs) using ionic liquids as modifiers and grape skins as carbon sources for the detection of Fe^3+^. Many classical fluorescent probe molecules—such as pyrene, perylene diimide (PDI), and their derivatives—exhibit strong fluorescence in dilute solutions [15,16,17]. However, when these molecules are present at high concentrations or form aggregates, their emission intensity dramatically decreases or even vanishes. This phenomenon, known as aggregation-caused quenching (ACQ), severely limits the use of such materials in solid-state light-emitting devices and aggregate-state sensing applications [18]. In contrast to ACQ systems, later studies revealed that numerous unconventional luminogens—often lacking extended π-conjugation—display negligible emission in their isolated molecular state but become highly emissive upon aggregation or cluster formation. This unusual behavior cannot be explained by traditional ACQ theory. To capture its underlying essence, researchers proposed the concept of clusterization-triggered emission (CTE). From the perspective of luminescent mechanisms, CTE molecules exhibit significant conformational stiffening and restricted intramolecular motion (RIM) upon aggregation or clustering, thereby effectively regulating their luminescent behavior [19,20]. Since the CTE system is susceptible to aggregated states and cluster structures in external environments, its luminescent intensity and wavelength will exhibit significant responses to environmental changes. Therefore, CTE molecules have an inherent advantage in achieving high-sensitivity and tunable fluorescent responses, providing theoretical feasibility for their design and application as fluorescent probes [21]. Qiu et al. [22] synthesized a cellulose-based fluorescent probe containing 1,4-dihydropyridine (DHPs) fluorophores based on water-soluble cellulose acetate succinate (CAA). This probe exhibits a clusterization-triggered emission (CTE) effect and can be used to detect Fe^3+^. Similarly, Xu et al. [23] demonstrated that gelatin shows a CTE effect arising from cluster formation and can also serve as a fluorescent probe for Fe^3+^ ions. In recent years, among various CTE materials, chitosan-based materials have attracted extensive attention due to their unique fluorescence properties.

Chitosan is a polysaccharide derived from crustaceans through the deacetylation of chitin. It has no toxic or side effects and good biocompatibility, biodegradability, hygroscopicity, and moisture retention [24]. Nevertheless, large amounts of crustacean and mollusk waste, such as crab shells, are discarded every year, causing serious environmental and resource issues [25]. Therefore, the efficient utilization and functionalization of chitosan derived from such waste have become an important research focus [26]. Beyond traditional applications, chitosan has emerged as a highly versatile platform across various advanced technological fields. Its excellent biocompatibility makes it an ideal choice for nanomedicine and therapeutic diagnostics, especially in the development of targeted drug delivery systems [27]. Furthermore, it has demonstrated significant potential in green nanofabrication, serving as a biodegradable resist for high-resolution patterning via atomic force nanolithography [28]. Due to the abundant amino and hydroxyl groups on the surfaces of chitosan molecular chains, chitosan also acts as an excellent host matrix for developing highly sensitive optical, fluorescent, and colorimetric biosensors [29]. Specifically in the realm of environmental monitoring, an increasing number of researchers are focusing on introducing fluorescent probe molecules via chemical modification of their functional groups to achieve ion detection, providing novel insights into the development of biomass-valorized fluorescent materials [30,31]. For example, Hu et al. [32] prepared a novel fluorescent probe with high sensitivity and selectivity for hypochlorite(ClO^−^) from chitosan via acylation and Suzuki coupling reactions. However, many of these reported fluorescent materials still suffer from issues such as toxicity, complex synthesis, or poor environmental compatibility [33]. Therefore, it is highly necessary to develop a simple, sustainable, and highly sensitive chitosan-based CTE fluorescent probe for the detection of metal ions.

**Figure 1:**
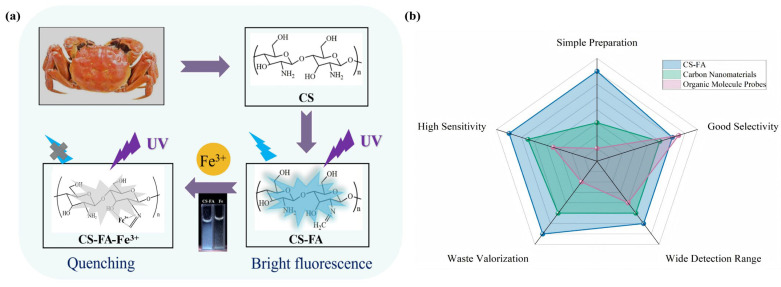
(**a**) Schematic diagram of the preparation process for the CS-FA fluorescent probe. (**b**) Performance comparison of different samples.

In this study, we developed a novel biomass-based fluorescent probe (CS-FA) through formaldehyde cross-linking for highly selective detection of Fe^3+^ ions. As shown in Figure 1a, the chitosan precursor is first extracted from natural crab shells through a series of continuous processes: demineralization, deproteinization, and deacetylation. Subsequently, a simple condensation reaction was performed between chitosan and formaldehyde to prepare a CS-FA probe. This structural cross-linking successfully activated the CTE mechanism, endowing the probe with strong blue fluorescence under UV excitation. In addition, the CS-FA probe exhibits excellent specificity and sensitivity in recognizing Fe^3+^. As shown in Figure 1b, this probe offers several advantages, including waste valorization, a simple preparation process, good selectivity, high sensitivity, and a wide detection range. In summary, this work not only provides a highly promising and waste-to-resource strategy for efficient iron ion monitoring but also broadens the high-value utilization pathways of marine biomass waste [34].

## 2. Experimental Section

### 2.1. Materials

The crab shells were obtained from a seafood market. FeCl_3_ (98%) was purchased from Shanghai Nadingshenghua Technology Co., Ltd. (Shanghai, China). KCl (99.8%) was procured from Shandong Keyuan Biochemical Co., Ltd. (Jinan, China). Other reagents in this experiment were of analytical grade. HCHO and HCl were procured from Xilong Technology Co., Ltd. (Shenzhen, China) and Modern Oriental (Beijing) Technology Development Co., Ltd. (Beijing, China), respectively. Ferric chloride hexahydrate was provided by Shanghai Nade Biochemical Technology Co., Ltd. (Shanghai, China). NaClO_2_ (80%), NaOH (98%), C_4_H_11_NO_3_ (99.9%), CuCl_2_·2H_2_O (analytical purity) and others were all purchased from Shanghai Macklin Biochemical Technology Co., Ltd. (Shanghai, China).

### 2.2. Synthesis of CS-FA

The preparation of CS-FA is carried out in two steps. In the first step, CS is synthesized. First, a certain amount of crab shells is washed, dried, and ground into powder. The crab shell powder (3 g) is placed in a sodium chlorite solution for decolorization, thereby altering its structure. Then, the pretreated crab shell powder is added to a 1.0 mol/L hydrochloric acid solution and reacted at 75 °C for two hours to remove mineral components. Subsequently, the sample is washed and dried at 60 °C to constant weight. Next, the washed sample is added to a sodium hydroxide solution and heated to 65 °C for 3 h to hydrolyze and remove fats and proteins. After cooling, it is washed until the filtrate is neutral, and the dried product is crude chitin. Finally, the crude chitin is placed in a high-concentration sodium hydroxide solution and reacted at 100 °C for six hours to obtain chitosan.

In the second step, the CS-FA probe was prepared. The CS (1 g) was dissolved in 20 mL of ethanol, and formaldehyde (2 mL) was added to the CS solution. The solution was then stirred at room temperature for 12 h. After the reaction, the products were washed with deionized water and dried at 60 °C. The product was named CS-FA.

While the utilization of crab shells provides a renewable pathway for probe synthesis, the comprehensive sustainability of this process requires an objective evaluation. The initial extraction of chitin and its deacetylation to chitosan involve necessary harsh chemical treatments, consuming HCl, concentrated NaOH, and NaClO_2_. Consequently, this process generates chemical waste that requires proper neutralization. Furthermore, although formaldehyde is a toxic precursor, it functions exclusively as a cross-linking agent in our synthesis, largely consumed during the condensation reaction to form stable Schiff base linkages. Extensive washing of the final CS-FA products with deionized water effectively removes unreacted formaldehyde residues. Therefore, the toxicity is primarily confined to the synthesis stage, while the final polymeric probe offers an effective “waste-to-resource” strategy.

### 2.3. CS-FA Fluorescent Probe for Fe^3+^ Detection

Prepare metal ions (Fe^3+^, Cu^2+^, Na^+^, K^+^, Ca^2+^, Zn^2+^, Ni^2+^, Mg^2+^, Co^2+^, and Mn^2+^) at a concentration of 0.05 mol/L, dilute them to 100 µM. Add 0.5 mL of Tris-HAc buffer solution (pH = 7.4), 0.4 mL of CS-FA probe solution (2 mg/mL), 0.1 mL of ultrapure water, and 3 mL of 100 µM metal ion stock solution, respectively. React the mixture at 25 °C for 10 min, then measure its fluorescence spectrum.

A 0.05 mol/L FeCl_3_ stock solution was prepared and diluted to concentrations ranging from 10 µM to 100 µM. The solutions were mixed with the CS-FA probe and incubated at 25 °C for 10 min, followed by fluorescence spectroscopy.

### 2.4. Characterization Methods

The prepared samples were observed using a scanning electron microscope (SEM, JSM-7610FPlus, JEOL, Tokyo, Japan). Before SEM observation, the samples were fixed onto a conductive adhesive and coated with a thin layer of gold using a sputter coater to eliminate charging effects and improve image quality. SEM micrographs were captured using a secondary electron (SE) detector at an accelerating voltage of 20.0 kV. Surface functional group composition was analyzed by a Fourier Transform Infrared Spectrometer (FT-IR, TENSOR II, Bruker, Ettlingen, Germany). The spectra were recorded over a range of 500–4000 cm^−1^. Phase composition was acquired with an X-ray diffractometer (XRD, D8 Advance, Bruker AXS, Karlsruhe, Germany). We collected high-quality XRD data for Rietveld refinement over the 5–80° range, with a step size of 0.02° and a 2 s per step delay. Photoluminescence emission (PL) and photoluminescence excitation (PLE) characterization were performed using a fluorescence spectrophotometer (F-4700, Hitachi, Tokyo, Japan).

### 2.5. Data Processing and Statistics

Data processing, linear fitting, and plotting were performed using Origin 2021. Microsoft Excel 2019 was used for basic data management and calculating the mean values and standard deviations (SD). The quantitative detection experiments were performed in triplicate using the same batch of fluorescent probes.

## 3. Results and Discussion

### 3.1. Characterization of CS-FA

**Figure 2:**
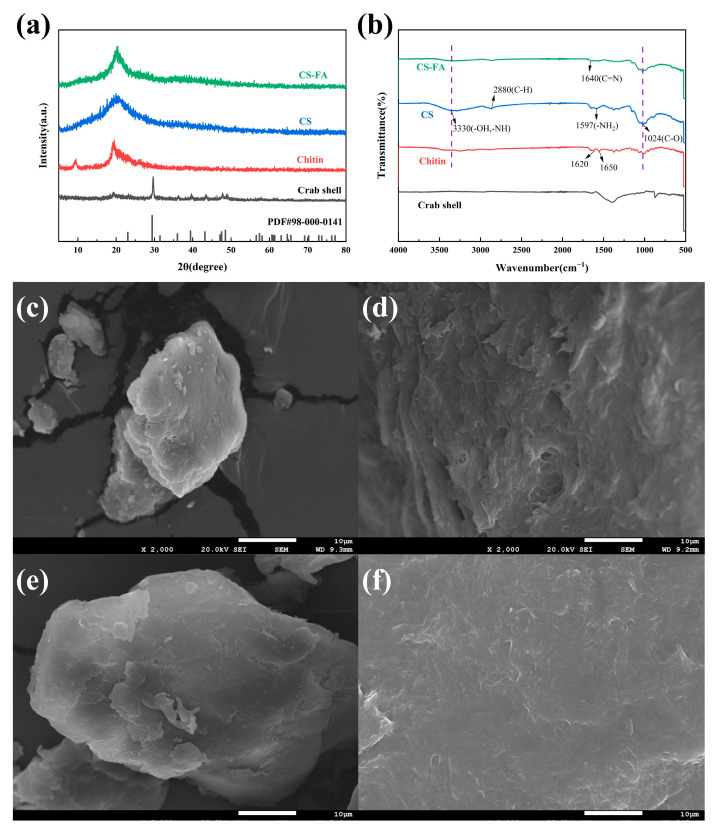
XRD (**a**) and FTIR (**b**) spectra of Crab Shell, chitin, CS, and CS-FA. SEM images of Crab Shell (**c**), chitin (**d**), CS (**e**), and CS-FA (**f**).

XRD was employed to characterize the phase composition of the samples (Figure 2a). For crab shells, a significant peak was observed at 2θ = 30.00°, indicating the presence of inorganic minerals, mainly calcium carbonate [35]. After acid-base treatment to obtain chitin, the significant mineral peaks basically disappeared, verifying the success of the demineralization process. Meanwhile, diffraction peaks appeared at 2θ = 10.00°, 20.00°, and 22.00°, corresponding to the typical α-chitin structure [36]. Only a broad diffraction peak at 2θ = 20.00° was observed, indicating successful deacetylation to form chitosan [26]. Compared with CS, the characteristic peaks of CS-FA near 2θ = 20.00° become sharper and more prominent. This structural transformation indicates that the condensation reaction with formaldehyde effectively restricts the mobility of chitosan chains, prompting them to rearrange into a more tightly packed, locally ordered structure [37,38].

As shown in Figure 2b, the sample components at different stages were characterized by infrared spectroscopy analysis. In the spectrum of crab shells, the absorption bands exhibit broad, dispersed, and low-intensity characteristics, reflecting their complex natural organic–inorganic composite characteristics [35]. In the spectrum of purified chitin, the distinct absorption peaks at 1620 cm^−1^ and 1550 cm^−1^ are attributed to the amide I band (C=O stretching vibration) and amide II band (N-H bending coupled with C-N stretching), respectively. Compared with the original crab shell, the significant enhancement of these characteristic peaks confirms the successful extraction of biopolymers and the effective removal of the composite matrix. After deacetylation to form CS, the peak appearing at 1597 cm^−1^ corresponds to the N-H bending vibration of the primary amine. Combined with the broadband at 3330 cm^−1^ (overlapping -OH and N-H stretching vibrations), this confirms the successful deacetylation and exposure of amino groups, providing convenient conditions for further formaldehyde modification. Finally, in the CS-FA spectrum, the new peak at 1640 cm^−1^ was mainly assigned to the C=N stretching vibration of the Schiff base [39]. This provides evidence for the successful condensation cross-linked reaction between amino groups and formaldehyde. Importantly, this cross-linking involves a multi-pathway chemistry, generating both initial Schiff-base linkages and subsequent methylene bridges [40,41]. This synergistic process creates a highly dense and rigid network that restricts intramolecular motion and forces electron-rich heteroatoms into close proximity, fundamentally activating the clusterization-triggered emission (CTE) mechanism.

The microscopic morphologies of crab shells, chitin, CS, and the CS-FA fluorescent probe were further observed by SEM. As shown in Figure 2c, the crab shell presented an irregular blocky structure with a rough surface. This is because chitin and protein combine to form a skeleton, and minerals, mainly calcium carbonate, are deposited on it [42]. As shown in Figure 2d–f, after extracting chitin, the subsequent deacetylation to generate chitosan (CS) and the cross-linking to prepare CS-FA probes did not result in significant changes in the macroscopic morphology of the material. Chitin, CS, and CS-FA all exhibit similar solid bulk structures. This observation demonstrates that the sequential chemical modifications occurred in situ, effectively preserving the basic structural framework of the natural biopolymer.

### 3.2. Fluorescence Properties of CS-FA

**Figure 3:**
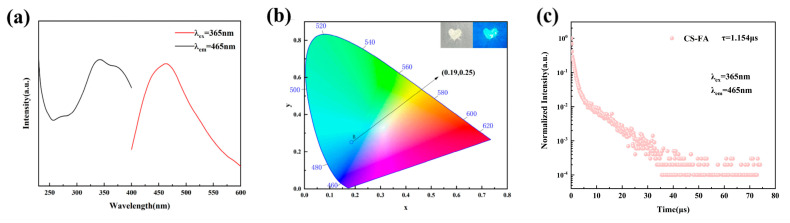
(**a**) The PLE and PL spectra of the CS-FA fluorescent probe. (**b**) The CIE chromaticity diagram of the CS-FA fluorescent probe. (**c**) The decay curves of the CS-FA fluorescent probe.

Figure 3a represents the excitation and emission spectra of the CS-FA fluorescent probe at room temperature. The CS-FA probe exhibits a broad emission peak in the 400–600 nm range, with a maximum at 465 nm under 365 nm excitation. This strong blue emission fundamentally originates from clusterization-triggered emission (CTE). The Schiff-base (C=N) cross-linking provides the highly rigid spatial framework required to restrict intramolecular motions, thereby actively forcing the electron-rich heteroatoms (N and O) into tight clustering to activate the robust fluorescence [38]. The CIE coordinates corresponding to the fluorescent probe (0.19, 0.25) are shown in Figure 3b. In addition, CS-FA appears colorless or pale yellow under sunlight and emits bright blue fluorescence under ultraviolet light. Figure 3c shows the decay curve of CS-FA monitored at 465 nm under 365 nm excitation. Generally, the curve exhibits a non-exponential behavior expressed by the following function:

(1) $$\tau =\frac{\int_{0}^{\infty }I(t)tdt}{\int_{0}^{\infty }I(t)dt}$$

where *t* and *I*(*t*) mean the time and corresponding emission intensity. Due to the complex and diverse microenvironments of the emissive clusters in CTE-active materials, the decay curve exhibits a non-exponential nature. Therefore, Equation (1) was used to obtain the calculated average luminescence lifetime (τ), which was determined to be 1.154 μs. This relatively long, microsecond-scale lifetime is typical of CTE-active materials, which commonly exhibit prolonged emission lifetimes due to the effective stabilization of cluster excitons [38].

### 3.3. Detection of Metal Ions

**Figure 4:**
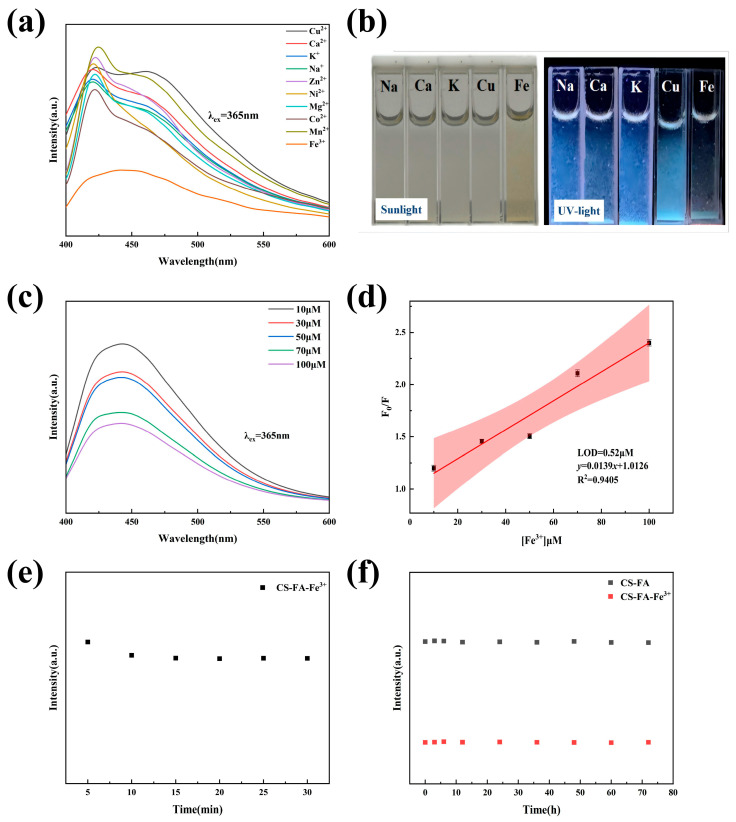
(**a**) The PL spectra of CS-FA after adding various ions. (**b**) Photograph of CS-FA after adding various ions under sunlight and UV-light (365 nm). (**c**) Fluorescence spectra of the probe upon progressive titration with Fe^3+^. (**d**) Linear calibration plot of F_0_/F versus Fe^3+^ concentration. Error bars represent the standard deviations obtained from three independent measurements (*n* = 3), and the pink shaded area indicates the 95% confidence band for the linear regression model. (**e**) Fluorescence intensity of CS-FA monitored at 465 nm for different times after the addition of 100 µM Fe^3+^. (**f**) Fluorescence intensity of CS-FA with and without Fe^3+^ at other times.

Chitosan and its derivatives have recognized multifunctional and powerful chelating abilities for a wide range of heavy metals and transition metal ions [43]. To investigate the specific recognition capability of the CS-FA probe, its fluorescence responses to various metal ions (Ca^2+^, Cu^2+^, K^+^, Na^+^, Zn^2+^, Ni^2+^, Mg^2+^, Co^2+^, and Mn^2+^) were studied. A certain amount of the CS-FA solution was added to solutions of different metal ions at the same concentration, and the resulting fluorescence was detected, as shown in Figure 4a. Figure 4b shows the photos of the CS-FA probe in solutions containing representative competing ions under sunlight and ultraviolet light. Under ultraviolet light, Cu^2+^, K^+^, Na^+^, and Ca^2+^ did not have an apparent quenching effect on the fluorescence, and blue fluorescence was emitted. However, the fluorescence of the probe solution containing Fe^3+^ was quenched, and the mixed solution appeared light yellow under natural light. Observations from both aspects confirmed that the CS-FA probe shows high selectivity for Fe^3+^.

Figure 4c shows the PL spectra of CS-FA at different Fe^3+^ concentrations under 365 nm excitation and explores the probe’s sensitivity to Fe^3+^. The fluorescence intensity decreases gradually as the Fe^3+^ concentration increases. This concentration-dependent quenching phenomenon highlights the high sensitivity of the CS-FA probe to Fe^3+^. In addition, a linear relationship was observed between the fluorescence intensity ratio and Fe^3+^ concentration (Figure 4d). The regression equation obtained by linear fitting is *y* = 0.0139*x* + 1.0126, with a correlation coefficient R^2^ = 0.9405. According to the IUPAC-recommended criterion (LOD = 3σ/S), the theoretical limit of detection for Fe^3+^ was determined to be 0.52 μM, where σ represents the standard deviation of the blank response (n = 10) and S denotes the slope of the fitting curve. This result highlights its excellent analytical performance in Fe^3+^ detection. Figure 4e shows the fluorescence intensity of CS-FA at different times after addition to the Fe^3+^ solution. The fluorescence intensity of CS-FA-Fe^3+^ remained basically unchanged within 30 min, indicating that the quenched state reached stability rapidly. Figure 4f shows the change in the fluorescence intensity of the CS-FA solution in the presence and absence of Fe^3+^. The fluorescence intensity was monitored for 72 h and remained stable, with no significant attenuation over time. Moreover, after adding Fe^3+^, the fluorescence intensity decreased by around 40%, yet the system remained stable.

As shown in Table 1, the CS-FA probe demonstrates highly competitive analytical performance compared to other reported methods, particularly benefiting from its simple preparation and applicability in near-neutral aqueous buffer (pH = 7.4). Importantly, compared to conventional probes that often require expensive precursors, the CS-FA probe achieves this comparable detection capability through a far more economically viable “waste-to-resource” pathway.

**Figure 5:**
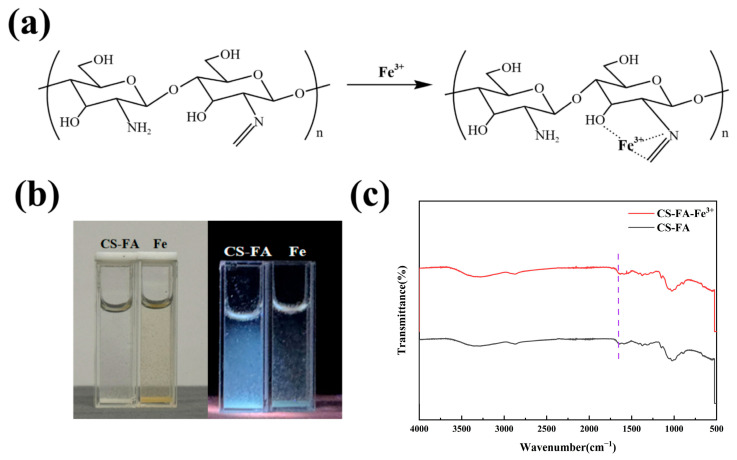
(**a**) The mechanism for the detection of Fe^3+^ ions. (**b**) Photograph of CS-FA after adding Fe^3+^ under sunlight and UV light (365 nm). (**c**) The FTIR spectra of the CS-FA and CS-FA-Fe^3+^. The dashed line indicates the position of the absorption band near 1640 cm^−1^.

Figure 5a illustrates the response mechanism of the CS-FA probe to Fe^3+^. It is worth noting that under near-neutral conditions (pH = 7.4), the Tris-HAc buffer transiently stabilizes Fe^3+^, while the strong multidentate chelation of CS-FA effectively outcompetes the buffer to capture the Fe^3+^ ions. Fe^3+^ interacts with oxygen and nitrogen atoms in the CS-FA framework, mainly via hydroxyl and imine-like functional groups, forming a stable coordination complex [50]. This coordination perturbs the local electronic environment and facilitates non-radiative energy transfer, thereby quenching fluorescence. The CS-FA probe in an aqueous solution is colorless under natural light. After adding Fe^3+^, it turns slightly yellow. It emits strong fluorescence under ultraviolet irradiation, whereas Fe^3+^ significantly quenches it (Figure 5b). The FTIR spectra before and after adding Fe^3+^ are shown in Figure 5c. Upon addition of Fe^3+^, the absorption band near 1640 cm^−1^ shifts to lower wavenumbers. Therefore, the coordination of the nitrogen atom’s lone pair electrons to Fe^3+^ reduces the bond order of the C=N bond and alters its vibrational frequency [51]. Consequently, these structural variations serve as spectroscopic evidence for the formation of a stable ground-state complex. As reported in similar chitosan-based and CTE-active sensing systems, the formation of such specific coordination complexes inherently restricts spatial emissive interactions and induces non-radiative decay [30,43,50]. From a photophysical perspective, this suggests that a static quenching mechanism, intrinsically driven by specific chemical coordination, may play a predominant role in the observed fluorescence reduction.

## 4. Conclusions

In summary, this study successfully designed and prepared a novel biomass-based fluorescent probe (CS-FA) with a stable cross-linked structure through a simple condensation reaction between chitosan and formaldehyde. We activated the clusterization-triggered emission (CTE) mechanism by spatially clustering electron-rich heteroatoms within the cross-linked structure. This structural engineering effectively limits non-radiative exciton dissipation, endowing the CS-FA probe with strong, stable blue fluorescence at 465 nm and characteristic CIE coordinates. Importantly, the CS-FA probe can serve as a highly selective and sensitive chemical sensor for the detection of Fe^3+^ in aqueous media. Mechanistic studies have shown that the specific multidentate coordination between Fe^3+^ and nitrogen/oxygen atoms in the CS-FA skeleton effectively disrupts the CTE effect and triggers a significant fluorescence quenching response. This probe exhibits excellent quantitative analysis capabilities, with a linear detection range of 10–100 µM and a minimum detection limit (LOD) as low as 0.52 µM. Compared with traditional synthetic fluorescent dyes, the CS-FA probe offers competitive advantages, including a simple preparation process, a highly stable structure, and the effective utilization of biological waste. Ultimately, this work provides a reliable, low-cost platform for monitoring Fe^3+^ in environmental systems. Furthermore, immobilizing the CS-FA probe onto solid supports represents a promising direction for future research, which would facilitate the development of portable and reusable sensing devices. This study promotes the development of new biomass fluorescence sensors and opens up new avenues for the high-value-added utilization of marine waste-derived materials.

## Figures and Tables

**Table 1:** Comparison of the detection of Fe^3+^ by different probes.

Fluorescent Material	LOD (μM)	Range (μM)	Sensing Medium	Response Time	Ref.
coumarin-3-carboxylic acid and 3-hydroxyaminomethane	1.683	0–500	H_2_O (pH = 4–9)	30 s	[44]
nitrogen and sulfur co-doped carbon dots	0.67	10–90	Aqueous solution	Rapid (not specified)	[45]
IL-CQDs	13.68	0–300	Ultra-pure water	30 min	[46]
benzothiazole biphenyl fluorophore	1.16	0–200	PBS buffer (pH 5.0)/ethanol (49:1, *v*/*v*)	9–10 min	[47]
Oxalis corniculata-derived carbon dots	0.73	10–50	Aqueous solution	Rapid (not specified)	[48]
SiO_2_@Nap material	1.31	100–900	Aqueous medium (pH = 7)	Not specified	[49]
CS-FA	0.52	10–100	Tris-HAc buffer solution (pH = 7.4)	10 min	This work

## Data Availability

The original contributions presented in this study are included in the article. Further inquiries can be directed to the corresponding author.
